# Advances in Single-Particle Electron Cryomicroscopy Structure Determination applied to Sub-tomogram Averaging

**DOI:** 10.1016/j.str.2015.06.026

**Published:** 2015-09-01

**Authors:** Tanmay A.M. Bharat, Christopher J. Russo, Jan Löwe, Lori A. Passmore, Sjors H.W. Scheres

**Affiliations:** 1 Structural Studies Division, MRC Laboratory of Molecular Biology, Francis Crick Avenue, Cambridge CB2 0QH, UK

## Abstract

Recent innovations in specimen preparation, data collection, and image processing have led to improved structure determination using single-particle electron cryomicroscopy (cryo-EM). Here we explore some of these advances to improve structures determined using electron cryotomography (cryo-ET) and sub-tomogram averaging. We implement a new three-dimensional model for the contrast transfer function, and use this in a regularized likelihood optimization algorithm as implemented in the RELION program. Using direct electron detector data, we apply both single-particle analysis and sub-tomogram averaging to analyze radiation-induced movements of the specimen. As in single-particle cryo-EM, we find that significant sample movements occur during tomographic data acquisition, and that these movements are substantially reduced through the use of ultrastable gold substrates. We obtain a sub-nanometer resolution structure of the hepatitis B capsid, and show that reducing radiation-induced specimen movement may be central to attempts at further improving tomogram quality and resolution.

## Highlights

• The Bayesian approach in RELION is extended to sub-tomogram averaging
• A new 3D CTF and missing-wedge model for sub-tomogram averaging is proposed
• Ultrastable gold supports reduce radiation-induced motion in tomography tilt series
• Using the above, an 8 Å structure of hepatitis B capsid from cryo-ET is presented

## Teaser

Bharat et al. have harnessed recent innovations in specimen preparation, data collection, and image processing from the field of cryo-EM single-particle analysis to improve electron cryotomography and sub-tomogram averaging.

## Introduction

High-resolution structure determination by single-particle analysis of electron cryomicroscopy (cryo-EM) data is undergoing rapid progress. New direct electron detectors have yielded images with signal-to-noise ratios that are significantly better than were previously available on film (McMullan et al., 2009, 2014). Using direct electron detectors each exposure can be divided into multiple image frames, and these frames can be saved as a movie. These movies can be used to reduce the image blurring caused by radiation-induced specimen movements, which results in restoration of high-resolution features in the images (Brilot et al., 2012; Campbell et al., 2012; Fromm et al., 2015; Li et al., 2013; Scheres, 2014). Also, new substrates reduce specimen movement to improve image quality (Russo and Passmore, 2014a, 2014b). Added to these instrumentation developments, improvements in refinement algorithms have also made image alignment and classification more accurate, resulting in higher-resolution structures from lower amounts of cryo-EM data than was previously possible (Bai et al., 2013; Scheres, 2012a). This has led to a number of near-atomic resolution cryo-EM structures by single-particle analysis (e.g. Allegretti et al., 2014; Bartesaghi et al., 2014; Liao et al., 2013; Wong et al., 2014).

Single-particle analysis requires images of many identical copies of a macromolecule in different “views.” Each of these views corresponds to a two-dimensional (2D) projection of the Coulomb scattering potential (or cryo-EM density) of the particle. In the Fourier domain, the 2D projections are central slices through the three-dimensional (3D) Fourier transform of the potential, and the corresponding projection directions determine the orientations of these 2D slices. Each image must be aligned with respect to the 3D structure to precisely orient each 2D Fourier slice. Because radiation damage puts stringent limitations on the electron dose one can use for imaging, the signal-to-noise ratio in these images is low, and one needs to average over many images. Even with the new detectors, tens of thousands of asymmetric units are required to calculate a 3D reconstruction of the scattering potential at near-atomic resolution.

In some specimens, the molecule of interest cannot be isolated from its complex 3D environment, for example flagella (Lin et al., 2014), asymmetric viruses such as HIV-1 or herpesvirus (Briggs et al., 2009; Frank et al., 2015; Grünewald et al., 2003), purified parts of cells such as the nuclear pore complex (Beck et al., 2007), or even whole cells (Bharat et al., 2011; Tocheva et al., 2011). In these cases, it is not possible to record images of individual macromolecules as separated views. Instead, other molecules surround the target macromolecule, and in 2D cryo-EM images the target signal is superimposed with the signal originating from the environment. For these complex samples, electron cryotomography (cryo-ET) is an alternative method of 3D structure determination. In cryo-ET, multiple low-dose 2D cryo-EM images of the same specimen are collected as a tilt series, i.e. as multiple images at different tilt angles of the sample relative to the electron beam (Baumeister, 2002). These images are then used to obtain a tomogram, which is a 3D reconstruction of the entire field of view. Because of the geometry of specimen holders, high-tilt images cannot be recorded, resulting in an empty region in the 3D Fourier transform of a tomogram. When the sample is tilted around a single axis, this region is wedge shaped and is often referred to as the missing wedge. Apart from specific artifacts that are caused by the missing wedge, we assume a tomogram is an accurate representation of the arrangement of the scattering potential of the specimen in 3D, and within a tomogram multiple copies of the same structure may be present.

3D averaging of repeated structures in tomograms, or sub-tomogram averaging, may further reduce noise and thus reveal higher-resolution information about those structures (Bartesaghi and Subramaniam, 2009; Briggs, 2013; Förster et al., 2005). Recent progress in cryo-ET data collection schemes with improved defocus stability over the entire tilt series, and new image-processing algorithms with contrast transfer function (CTF) correction, have allowed sub-nanometer resolution structures to be determined using sub-tomogram averaging (Schur et al., 2015). In addition, hybrid image-processing approaches have been described that use information from sub-tomogram averaging to guide single-particle analysis (Bartesaghi et al., 2012; Bharat et al., 2012, 2014). Nonetheless the highest-resolution structures obtained in sub-tomogram averaging are significantly lower in resolution than those obtained by single-particle analysis, and it is not clear why this is the case.

Since radiation damage limits the total electron dose that can be applied to the specimen (Henderson, 2015), each image of a tomographic tilt series is typically collected with a fraction of the dose applied in conventional single-particle cryo-EM images (1–5 e^−^/Å^2^ versus 25–30 e^−^/Å^2^). It is therefore more difficult to accurately estimate CTF parameters in tomography, particularly from high-tilt images, where the effective signal-to-noise ratio is very low (Eibauer et al., 2012). Overall, typical electron doses applied over the entire tilt series (40–100 e^−^/Å^2^) are higher than in conventional single-particle analysis (25–30 e^−^/Å^2^). This extra electron dose destroys high-resolution features in the specimen, and ultimately limits the attainable resolution in sub-tomogram averaging. Apart from these limits imposed by radiation damage, the algorithms used for sub-tomogram averaging may align and classify the individual particles less accurately than analogous single-particle algorithms. Furthermore, the motion of the specimen during irradiation is likely another factor limiting tomogram quality (Brilot et al., 2012; Campbell et al., 2012; Russo and Passmore, 2014b).

In this study, we sought to explore limitations in sub-tomogram averaging through the application of developments that have recently advanced resolution in single-particle analysis. The empirical Bayesian approach to image processing in the RELION program (Scheres, 2012a, 2014b), which has proved to be powerful for classification and high-resolution refinement of single-particle data (Bai et al., 2015), was extended for sub-tomogram averaging refinement. A new 3D CTF model was developed within the Bayesian framework of RELION, which, in line with standard practice in single-particle analysis, allows full CTF phase and amplitude correction. The parameters for this 3D CTF model, which also compensate for the missing wedge, were estimated using an extended tilt series data acquisition scheme on a direct electron detector. In addition, we performed experiments to show that radiation-induced specimen motion is an important limitation in tomogram quality, and used recently developed ultrastable gold substrates (Russo and Passmore, 2014b) to improve tomogram quality by reducing these motions.

## Results

### Approach

#### Image Processing

The image-processing approach to sub-tomogram averaging presented here builds directly on the empirical Bayesian approach to single-particle reconstruction presented previously (Scheres, 2012b; Scheres and Chen, 2012). The main differences are the three-dimensionality of the data, and the way the tomographic missing wedge and the CTF are treated simultaneously in a single 3D model. As before, the data model is formulated in the Fourier domain:

(Equation 1) $$X_{ij}={\text{CTF}3\text{D}}_{ij}{\left[R_{\phi }V_{k}\right]}_{j}+N_{ij}\text{,}$$

where

• *X*_*ij*_ is the *j*^th^ Fourier component of the 3D Fourier transform of the *i*^th^ experimental sub-tomogram, with *i* = 1, *…*, *N* and *j* = 1, …, *J*.
• CTF3D_*ij*_ is the *j*^th^ component of a 3D model in the Fourier domain that describes both the effects of the objective lens CTF on the 2D images in the tilt series, and the effects of the missing wedge. This model is described in more detail below; it is estimated outside the Bayesian framework.
• **R**_*ϕ*_ is a transformation that describes a combined rotation and translation *ϕ* in the real domain, i.e. a rotation and a phase shift in the Fourier domain. [**R**_*ϕ*_*V*_*k*_]_*j*_ represents the *j*^th^ Fourier component of a rotated reference (see below).
• *V*_*k*_ is the 3D Fourier transform of the *k*^th^ of *K* references, or underlying structures in the dataset. Values of *K* > 1 may be used to describe structural heterogeneity in the data, and *K* is an input parameter. All *J* Fourier components *V*_*kj*_ of all *K* references are assumed to be independent, zero-mean, and Gaussian distributed with a variance $\tau_{kj}^{2}$.
• *N*_*ij*_ is an instance of noise in the 3D Fourier domain. For all Fourier components of all *N* experimental sub-tomograms, this noise is assumed to be independent, zero-mean, and Gaussian distributed with variance $\sigma_{ij}^{2}$.

As in the case of single-particle reconstruction, a regularized likelihood function is optimized, and one marginalizes over the unknown orientations *ϕ* and the class assignments *k* of all sub-tomograms. The optimization target thus becomes:

(Equation 2) $$\left\{\prod_{i=1}^{N}\int_{\phi }\sum_{k=1}^{K}P\left(X_{i}|k,\phi ,\Theta \right)P\left(k,\phi |\Theta \right)d\phi \right\}\cdot P\left(\Theta \right)\text{,}$$

where *P*(*X*_*i*_|*k*,*ϕ*,Θ) is calculated as a multiplication of *J* Gaussians, centered on CTF3D_*ij*_[**R**_*ϕ*_*V*_*k*_]_*j*_ and with variance $\sigma_{ij}^{2}$, for each Fourier component *X*_*ij*_. Likewise, the prior *P*(Θ) is calculated as a multiplication of Gaussians, each centered at zero and with variance $\tau_{kj}^{2}$, for all *J* Fourier components *V*_*kj*_ of all *K* references. *P*(*k*,*ϕ*|Θ) expresses prior information about the distributions of *ϕ* and *k*. These priors were described for the single-particle case (Scheres, 2012a; Sigworth, 1998) and are not repeated here.

Optimization of the regularized likelihood target in Equation 2 using the expectation maximization algorithm (Dempster et al., 1977) is analogous to the single-particle case (Scheres, 2012b). The resulting iterative algorithm becomes:

(Equation 3) $$V_{kj}^{\left(n+1\right)}=\frac{\sum_{i=1}^{N}\int_{\phi }{\text{Γ}}_{i\phi }^{\left(n\right)}\frac{\text{CTF}3{\text{D}}_{ij}}{\sigma_{ij}^{2}}{\left[R_{\phi }^{T}X_{i}\right]}_{j}d\phi }{\sum_{i=1}^{N}\int_{\phi }{\text{Γ}}_{i\phi }^{\left(n\right)}\frac{\text{CTF}3{\text{D}}_{ij}^{2}}{\sigma_{ij}^{2}}d\phi +\frac{1}{\tau_{kj}^{2}}},$$

(Equation 4) $$\sigma_{ij}^{2^{\left(n+1\right)}}=\frac{1}{2}\int_{\phi }{\text{Γ}}_{i\phi }^{\left(n\right)}\|X_{ij}-\text{CTF}3{\text{D}}_{ij}{\left[R_{\phi }X_{i}\right]}_{j}{\|}^{2}d\phi ,$$

(Equation 5) $$\tau_{kj}^{2^{\left(n+1\right)}}=\frac{1}{2}{\left\|V_{kj}^{\left(n\right)}\right\|}^{2}\text{,}$$

where (*n*) is the iteration number, and ${\text{ Γ}}_{ik\phi }^{\left(n\right)}$ is the posterior probability of class assignment *k* and orientation assignment *ϕ* for the *i*^th^ sub-tomogram. In practice, values for $\sigma_{ij}^{2}$ and $\tau_{kj}^{2}$ are calculated as averages in equidistant shells from the origin in the Fourier transform, i.e. in a 1D vector as a function of resolution.

In the single-particle approach, the iterative algorithm is started by providing a single reference structure. In the test case presented in this study, the initial reference could also be generated de novo from the data by starting from random orientation assignments for all sub-tomograms. If *K* > 1, multiple starting models are generated by random division of the dataset in the first iteration. Initial estimates for $\tau_{kj}^{2}$ and $\sigma_{ij}^{2}$ are calculated from power spectra of the initial reference and the experimental sub-tomograms, respectively.

This approach to sub-tomogram averaging is implemented in the open-source RELION program, and will be distributed freely in the 1.4 release. By re-using the existing implementation for single-particle analysis, the resulting program conveniently taps into recent developments for single-particle analysis. This provides direct access to the automated refinement procedure in RELION (called 3D auto-refine), which includes a procedure to estimate angular accuracies (Scheres, 2012b), and employs a gold standard procedure for refinement of two independent half-sets (Scheres and Chen, 2012). Also, the post-processing functions in RELION are directly applicable. These include auto-masking functionality, a procedure to estimate the effects of masking on the Fourier shell correlation (FSC) curve (Chen et al., 2013), and automated *B*–factor correction for those cases that extend significantly beyond 10 Å resolution (Rosenthal and Henderson, 2003). In addition, the particle extraction and normalization functionality of RELION was expanded to include 3D data, to facilitate entry into the RELION workflow starting from (x,y,z) coordinates (in RELION-specific STAR or in a plain three-column text format) and tomograms in the MRC format.

#### CTF Model

The aberrations of the objective lens of the microscope and the effects of taking underfocused images, which are approximated in the Fourier domain by the CTF, affect the 2D images that form the tilt series of a tomography experiment. CTF3D_*i*_ is obtained by calculating 2D CTFs of all the images in the tilt series, and placing them as central slices into a three-dimensional Fourier transform, where the orientation of the slices is determined by the tilt angles of the images in the series. For this, we use a modification of the reconstruction algorithm in the RELION package (Scheres, 2012b). This two-step algorithm is closely related to standard Fourier inversion reconstruction algorithms. First, one employs a straightforward tri-linear interpolation to place the 2D CTFs, which are sampled on a Cartesian grid, into a twice-oversampled 3D Cartesian grid (in the Fourier domain) of the CTF3D_*i*_ model. Then, instead of dividing the resulting weighted sum of the 2D CTFs in the 3D transform by the summation of all the weights from the tri-linear interpolation (Ω), an iterative gridding algorithm is used to calculate weights *W* for all 3D Fourier terms:

(Equation 6) $$W^{\text{new}}=\frac{W^{\text{old}}}{\left(\Omega W^{\text{old}}\right)⊗\Psi }\text{,}$$

where *W* is initialized to 1 for all Fourier components, and *Ψ* is a Kaiser-Bessel kernel that spreads over several Fourier components. Typically ten iterations of Equation 6 are performed. By multiplying the weighted sum of the 2D CTFs in the 3D transform from the first step with the resulting *W* terms from the second step, one aims to correct for the non-uniform sampling of the transform (Pipe and Menon, 1999).

**Figure 1:**
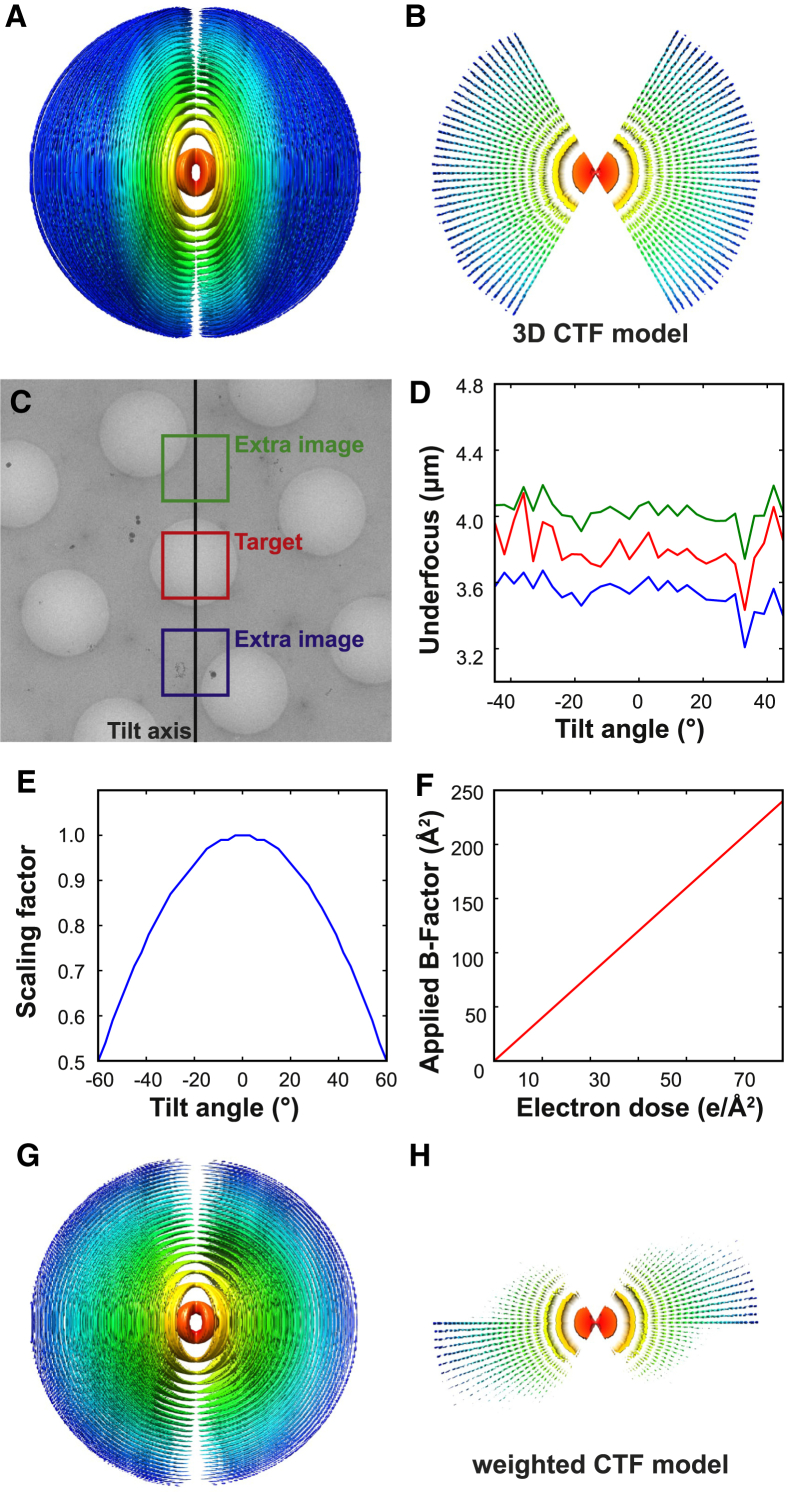
3D CTF Model and CTF Estimation (A) An isosurface view of the 3D CTF model used. The volume has been pseudo-colored based on radius (or resolution in Fourier space). Red indicates low resolution while blue indicates high resolution. The 3D CTF model is made of a series of 2D slices that represent 2D CTFs of each image of the tilt series. (B) An orthogonal view of (A) with only the central slice shown. (C) A low-magnification micrograph showing the extended tilt series acquisition used in this study. Two additional images were acquired to estimate the CTF parameters in the target region of interest. (D) A plot of estimated defoci in each of the three regions shown in (C) at different tilt angles. The difference between the green and the blue curves might be caused by an inclination of the sample with respect to the tilt axis. The diameter of holes in the micrograph is 2 µm. (E) Tilt angle-dependent scaling factor applied to weight the 3D CTF model. The multiplicative factor is equal to the cosine of the tilt angle, and scales the entire CTF curve downward. (F) Dose-dependent *B*–factor applied to the CTF model. The slope of the linear curve was determined empirically from a previous single-particle analysis report (Scheres, 2014). (G and H) Isosurface view (G) and central slice (H) of the final weighted 3D CTF model.

Figures 1A and 1B show the result for a typical CTF3D_*i*_. This function not only expresses information about the CTF modulations for each of the images in the tilt series (which, as explained in the next section, may be estimated as a function of the position of each particle in the tomogram). It also expresses information about the sampling of the tilt angle and (thus) the extent of the missing wedge. In fact, by expressing the missing wedge as a large region of zeros in CTF3D_*i*_ and by incorporating CTF3D_*i*_ inside the 3D Wiener filter in Equation 3, missing-wedge correction is done in the same manner as correction for zero-crossings in the CTFs. Intuitively, this means that differently oriented particles will have different missing regions in the Fourier domain, much like particles with different defoci have different zero-crossings in their CTFs.

One can interpret CTF3D_*i*_ as a function that describes the information transfer from a true molecular structure inside the microscope to each of the individual 3D Fourier components of the *i*^th^ experimental sub-tomogram. One caveat here is that the algorithm used for the tomogram reconstruction may be different from the algorithm used to generate CTF3D_*i*_. In the experiments described in this paper, we used weighted back-projection in Tomo3D (Agulleiro and Fernandez, 2011) to reconstruct the tomograms, and the Fourier inversion algorithm in RELION for reconstructing CTF3D_*i*_. The weighted back-projection algorithm results in different delocalizations of the signal in the Fourier domain. Thereby, the information transfer is not strictly the same as for the RELION-based reconstruction of CTF3D_*i*_. One could in principle overcome this by modifying Tomo3D to perform a reconstruction of CTF3D_*i*_, and the workflow we present here allows for the incorporation of alternative reconstruction algorithms for CTF3D_*i*_. However, because we did not expect large differences in the final results, we did not attempt such modifications in this paper.

In the current implementation, generation of all CTF3D_*i*_ is performed using a customized python script outside the RELION workflow, although this script does use the stand-alone reconstruction program in RELION (relion_reconstruct). The script may be downloaded from the RELION wiki pages (http://www.mrc-lmb.cam.ac.uk/relion).

#### CTF Estimation and Weighting

The new CTF model requires defocus estimates for each particle in all images of the tilt series. For this, we used an extended tilt series acquisition scheme (Eibauer et al., 2012) implemented in SerialEM (Mastronarde, 2005). Tomographic data were acquired on a direct electron K2-summit detector fitted behind a Gatan Quantum energy filter. Two additional, high-dose images were collected on either side of the region of interest along the tilt axis at the same distance (Figure 1C). The average defocus of each extra image was estimated using CTFFIND3 (Mindell and Grigorieff, 2003). The arithmetic mean of the estimated defoci for both extra images at each tilt was found to be a good estimate for the actual defocus value for the tilt series image (Figure 1D). Thus, this extended tilt series acquisition scheme provided CTF parameters for all images of the tilt series.

Sub-tomograms extracted from different parts of the image have a different defocus due to the tilting of the grid. Based on the position of the sub-tomogram in the reconstructed tomogram, we calculated the height difference of each particle in each image of the tilt series relative to the tilt axis. Adding the height difference to the average defocus of the tilt series image (calculated above) provided a local estimate of defocus for each sub-tomogram in each image of the tilt series.

Next, we sought to use the CTF model to appropriately weight the information present in the tilt series data. The effective thickness of the specimen increases at increasing tilt angle by a factor of 1/(cosine of the tilt angle). We postulated that the signal-to-noise ratio in the tilted images decreases proportionally to the increasing ice thickness. Thus, the 2D CTF function in slices corresponding to each tilted image in the 3D CTF model were damped down uniformly at all resolutions by multiplication with a factor equal to the cosine of the tilt angle (Figure 1E).

During data acquisition, the biological specimen is damaged by accumulated radiation. We assumed that the higher-resolution Fourier components decrease in amplitude first, and used a recently introduced radiation damage model for single-particle analysis (Scheres, 2014) to describe the fall-off of signal in the tilt series. We applied a linear dose-dependent *B*–factor to dampen the high-frequency components of images that were recorded later in the tilt series (Figure 1F). The combined tilt angle-dependent scale factor and the dose-dependent *B*–factor were used to obtain the final weighted CTF model (Figures 1G and 1H).

#### Limiting Radiation-Induced Motion with Ultrastable Gold Supports

Radiation-induced movement of the specimen is known to limit resolution in single-particle analysis. The blurring effects of this motion can be reduced using movie-processing algorithms, either by re-aligning entire micrographs (Li et al., 2013) or on a per-particle basis (Brilot et al., 2012; Scheres, 2014). For tomography, instead of one single exposure, the sample is irradiated multiple times in short exposures at different tilt angles of the specimen. The applied electron dose at each tilt angle can be fractionated in a movie, which can be used to compensate for radiation-induced motion within each of the short exposures. However, correcting for specimen movements that occur from one tilted image to the next is not straightforward. For this reason, in sub-tomogram averaging one cannot use per particle movie processing to improve the high-resolution signal as one would do in single-particle analysis. To study the effect of the beam on the specimen in tomography, tilt series data were collected on a DNA-origami test specimen (discussed below), and changes in the specimen that occurred during data acquisition were analyzed.

To better preserve high-resolution information in tomography, we used specimen supports that reduce radiation-induced motion of the specimen. Ultrastable supports made entirely of gold nearly eliminate substrate movement during irradiation (Russo and Passmore, 2014b). These ultrastable gold substrates were used in this study for tomography. They were fabricated so that the size of the hole containing vitreous ice was roughly the same as the field of view in the untilted image of the tilt series (∼1.2 μm diameter). The concentration of 10 nm gold fiducials was optimized to yield 8–12 markers in each hole.

### Hepatitis B Capsid as a Test Sample

We selected the hepatitis B virus (HBV) capsid protein as a test sample for our sub-tomogram averaging approach. This protein forms a closed shell with icosahedral symmetry. Symmetry was imposed for all refinements. By choosing a highly symmetric test specimen, many asymmetric units could be obtained in each tomogram, which allowed efficient testing of the limitations in the methods, rather than being limited by the size of the datasets. Moreover, the X-ray structure of this capsid is known (Wynne et al., 1999), which allows straightforward assessment of sub-tomogram reconstruction quality.

**Figure 2:**
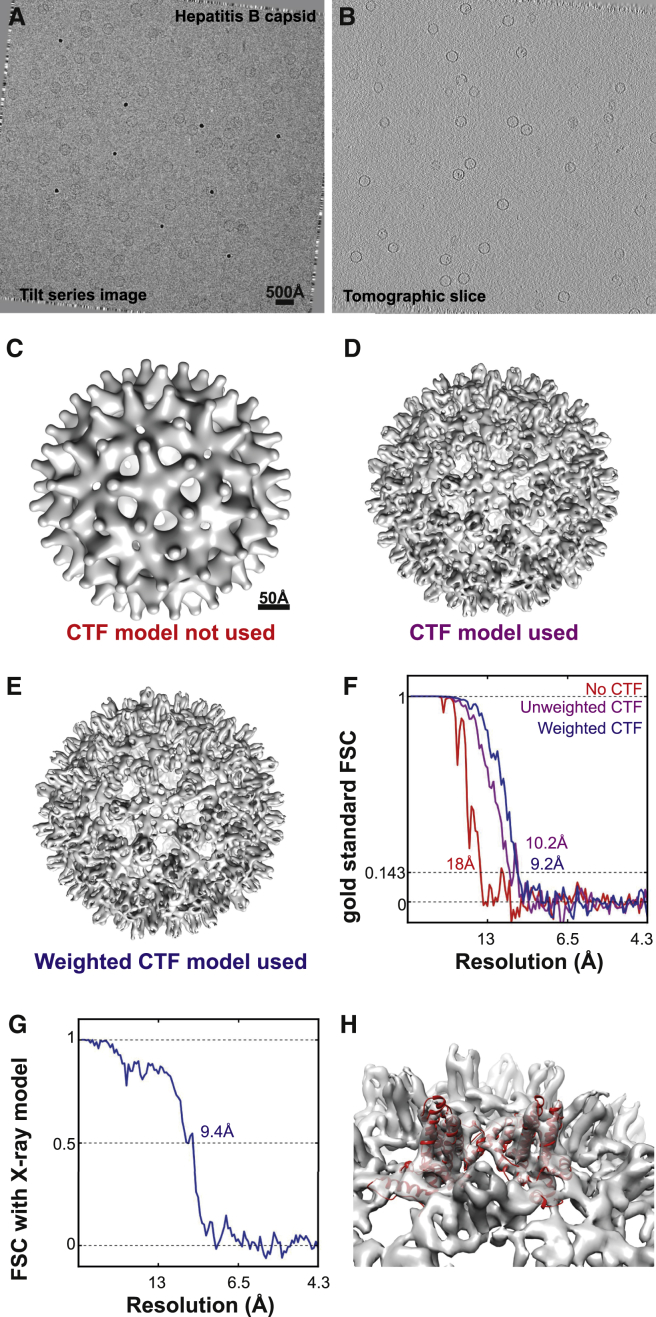
Maximum-Likelihood Refinement Using the 3D CTF Model (A) Representative image from a tilt series of the hepatitis B capsid sample. (B) Slice from a tomogram reconstructed from the tilt series data. The scale of (A) and (B) is the same. (C–E) Isosurface representations of the final volume of the refinement calculated (C) without CTF correction, (D) with the unweighted CTF model, and (E) with the weighted CTF model. The scale bar in (C) also applies to (D) and (E). (F) FSC curves showing the estimated resolution from the refinements. The same 1,851 HBV particles from 15 tomograms were used in all refinements. (G) FSC curve of the best model (from the refinement with weighted CTF model) against the X-ray structure. (H) The X-ray structure is shown fitted into the sub-tomogram averaging map, and secondary structure elements are clearly resolved. See also Figure S1.

We collected cryo-ET data on this sample (Figure 2A) and reconstructed tomograms from the data (Figure 2B). Tilt series alignment based on the gold fiducials was performed in IMOD (Kremer et al., 1996), and the actual tomogram reconstruction was performed using weighted back-projection in Tomo3D (Agulleiro and Fernandez, 2011). Particles were extracted from the reconstructed tomograms using the template-matching algorithm in the MolMatch software (Förster et al., 2005). To remove extracted sub-tomograms that did not correspond to HBV capsids, we conducted reference-free classification of the sub-tomograms in the initial dataset, using K = 2 in Equations 3–5 (Figures S1A and S1B). This classification readily allowed identification of real HBV capsid particles from the false positives that corresponded to 10-nm gold fiducials. Another efficient way to achieve a similar result is to project each sub-tomogram into a 2D image along the direction of the electron beam (Figure S1C), and to conduct reference-free classification of these 2D projections just as one would perform 2D class averaging in single-particle analysis (Figure S1D). This has been implemented as an easily accessible option in the particle extraction program of RELION. A similar method for the alignment of 3D data based on projections has been described previously (Yu et al., 2013), and we propose that using 2D classification of projected sub-tomograms could be a fast and efficient way to detect good particles for the next step of sub-tomogram averaging refinement.

For initial refinement tests with the identified HBV sub-tomograms, we used a 50-Å low-pass filtered version of the X-ray structure as a starting model. To investigate the effects of our new CTF model, we first conducted a refinement of the extracted particles without any CTF correction (Figure 2C). This refinement did not yield a high-resolution structure, and the final volume had no distinct features. Next, we conducted a refinement with the same parameters, with the unweighted 3D CTF model applied (Figures 1A and 1B). In this case, a 10.2 Å resolution structure was obtained (Figure 2D), showing that the 3D CTF model improves the algorithm.

Based on observed *B–*factors at the later stages of irradiation for a β-galactosidase sample (Scheres, 2014), we chose to use a linear increase of 4 Å^2^ in the *B*–factor with each electron per Å^2^ of accumulated electron dose (Figure 1F). Use of the tilt angle and electron dose-weighted 3D CTF model (Figures 1G and 1H) led to a further improvement in resolution to 9.2 Å (Figures 2E–2G). Clear rod-shaped densities for α–helices in the map confirmed the estimated resolution (Figure 2H).

**Figure 3:**
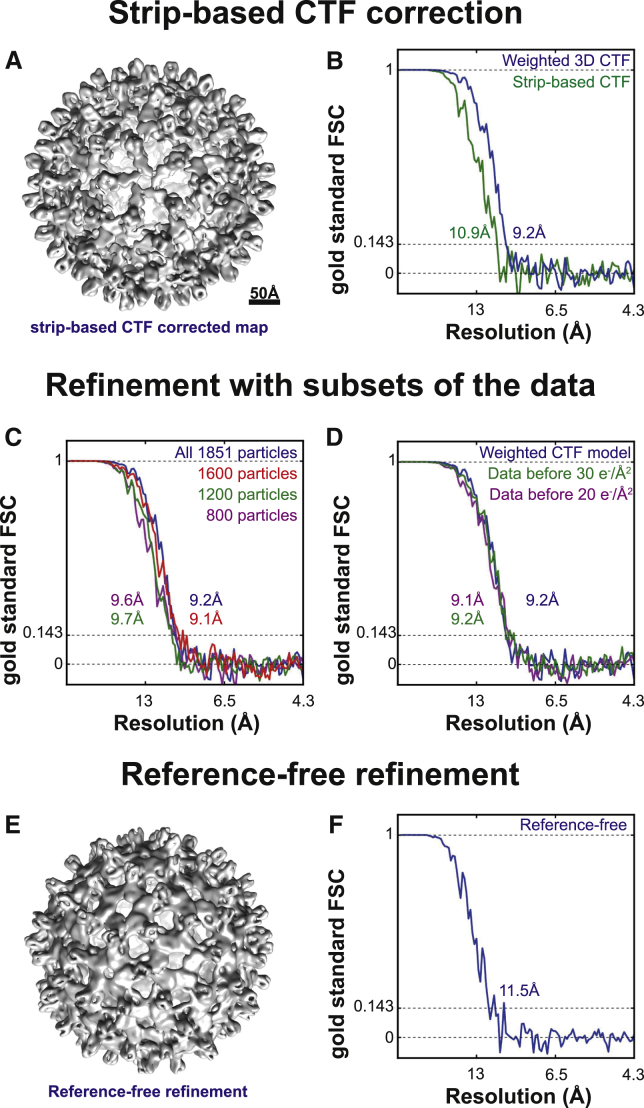
Refinements with Differing Parameters to Test the 3D CTF Model and to Probe the Limits of the Dataset (A) Using conventional strip-based CTF correction implemented in IMOD, automatic refinement in RELION yielded a 10.9 Å resolution map. Only phase flipping and no amplitude weighting was conducted in this CTF correction. (B) Compared with the refinement using our new 3D CTF model, the resolution obtained was lower. (C) Resolution of sub-tomogram averaging reconstruction could not be improved by adding more data or removing subsets of the data. Different sub-tomogram averaging refinements were conducted using random subsets of the data of different sizes. A similar resolution was obtained with fewer particles, showing that the refinement was not limited by the size of the dataset. (D) Part of the data that had been exposed to a cumulative electron dose of > 20 or 30 e^−^/Å^2^ was removed using the 3D CTF model. Compared with the full dataset in which 60 e^−^/Å^2^ had been applied to the specimen, no improvement was observed (the weighted 3D CTF model was used in all refinements). (E) Reference-free refinement using the same data as shown in Figure 2. Scale same as (A). (F) The resolution of the output, refined structure (11.5 Å) was not as high as in cases when a reference was used (9.2 Å).

To compare our new 3D CTF model with traditional, strip-based CTF correction schemes, we conducted the same refinement with data extracted from tomograms that had been CTF corrected in IMOD using strip-based phase flipping (Kremer et al., 1996). The obtained resolution (10.9 Å) was lower, showing that our new CTF model better reflects the information transfer in the sub-tomograms (Figures 3A and 3B). The highest-resolution sub-tomogram averaging structure (9.2 Å) could not be improved by the addition of more data, or by removing subsets of the data with the highest dose (Figures 3C and 3D).

Even though a low-pass filtered X-ray model was used for the above tests, a starting model could also be obtained from the data themselves by starting refinement from randomly assigned orientations to all sub-tomograms (Figures 3E and 3F). The resulting model was of a lower quality (11.5 Å) than the one obtained above, but using this as a starting model for a second refinement led to the same resolution as in the tests with an externally provided initial model. However, this approach for initial model generation may not be valid for all specimens. In particular for specimens with lower symmetry the problem is more complex, and we refer the reader to other studies focusing on this problem (Frank et al., 1986; Radermacher et al., 1986; Van Heel, 1987; Voss et al., 2010).

### Comparison of Sub-tomogram Averaging and Single-Particle Analysis on the Same Sample

**Figure 4:**
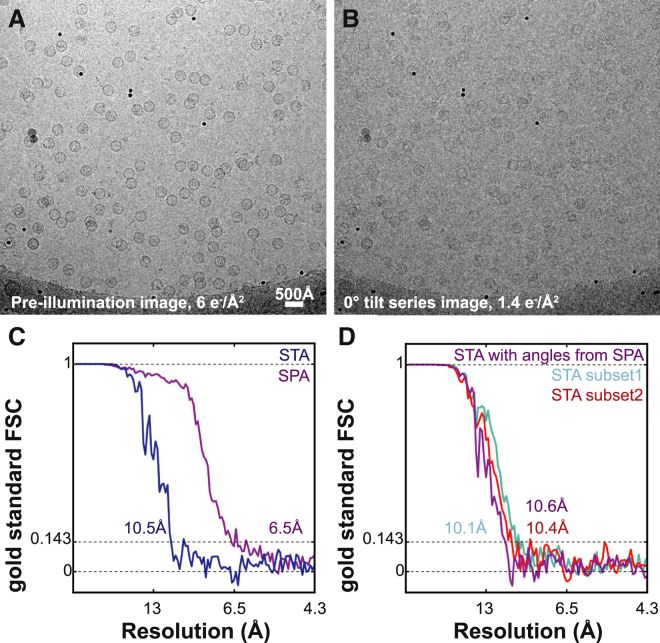
Pre-illumination for Comparing Sub-tomogram Averaging and Single-Particle Analysis (A) The sample was pre-illuminated with a dose of 6 e^−^/Å^2^ to record an image for single-particle analysis (SPA). (B) Immediately after this, a tilt series was collected of the same region with the same microscope parameters. The scale bar applies to (A) and (B). (C) Sub-tomogram averaging (STA) was conducted from the tomographic data, and single-particle analysis was conducted from the pre-illumination image using the same 1,501 HBV particles from 13 tomograms in both refinements. FSC curves of these refinements are shown. (D) Sub-tomogram averaging refinement of half of the particles (n = 751) that had the most similar alignment parameters in both refinements (denoted as subset 1, also see Figure S2) were compared with the other half (subset 2, n = 750) that had the most different alignment parameters. There was no significant difference between the resolutions obtained in both refinements. Furthermore, applying Euler angles obtained from single-particle analysis to the entire dataset (of 1,501 particles) did not lead to an improvement in sub-tomogram averaging (purple curve). See also Figure S2.

Our best resolved sub-tomogram averaging map (9.2 Å) remained far from limits imposed by the Nyquist frequency (4.3 Å). As a next test, we decided to compare sub-tomogram averaging and single-particle analysis refinements for the same particles. For this we pre-exposed the HBV capsid sample, collecting a conventional 2D cryo-EM image with a 6 e^−^/Å^2^ dose (Figure 4A), then acquired a tilt series of the same region (Figure 4B) with a dose of 60 e^−^/Å^2^. We used the tilt series data for tomographic reconstruction and extracted 1,501 HBV sub-tomograms for sub-tomogram averaging. The same particles were also selected from the pre-illuminated images for conventional single-particle analysis refinement. Sub-tomogram averaging produced a map at 10.5 Å resolution (Figure 4C, blue curve), while single-particle analysis refinement yielded a 6.5-Å resolution map (Figure 4C, purple curve).

The difference in resolution between the two structures calculated from pre-illuminated images and tomographic tilt series could be due to poor orientational assignments in the sub-tomogram averaging refinement. To test this, we calculated pairwise Euler angle differences between the two refinements for each particle (Figure S2), and divided the sub-tomograms into two subsets. Subset 1 contained half of the sub-tomograms (751 particles) that were assigned most similar angles in both sub-tomogram averaging and single-particle analysis refinements. Subset 2 contained the other half of the dataset (750 particles), i.e. sub-tomograms that were aligned with the most different angles in the sub-tomogram averaging and single-particle analysis refinements. Sub-tomogram averaging refinements of these two subsets yielded maps with similar resolutions (Figure 4D), indicating that alignment quality was not the limiting factor of the sub-tomogram averaging map. To confirm this, we also calculated an average of all sub-tomograms whereby we assigned the angles determined in the single-particle analysis refinement (Figure 4D). Again, this procedure did not lead to any improvement in resolution.

### Radiation-Induced Motion in Electron Cryotomography

To test the importance of radiation-induced specimen motions in tomography, we analyzed the motion of particles during tilt series data collection. Our aim was to independently track and orient each particle in each image of the aligned tilt series, and compare its position and orientation with that in the final reconstructed tomogram. For these tests we selected DNA-origami molecules that are large (6 MDa), high-contrast particles with a specifically designed shape to facilitate alignment, i.e. with strong, low-frequency components (Bai et al., 2012). Furthermore, these particles are asymmetric (i.e. they do not possess any symmetry-related views), making comparison between different orientations in our tilt-pair-like analysis (see below) easier. We collected tilt series at a relatively high defocus (−7 μm) and fluence (70 e^−^/Å^2^), and avoided tilt angles above 30° (Figure S3A). The alignment of the tilt series was conducted using gold fiducials as markers, and did not rely on the DNA-origami particles.

Sub-tomograms were selected manually from the tomographic reconstructions (Figure S3B), and these particles were carefully tracked to the individual particles in the aligned tilt series images (Figure S3A). Using an 11-Å resolution cryo-EM structure of this DNA-origami molecule (EMD-2210) as a reference, we assigned Euler angles and shifts to the sub-tomograms as well as the particles in each image of the tilt series. We compared the assigned Euler angles in the sub-tomogram averaging and single-particle analysis alignments in a tilt-pair-like analysis (Rosenthal and Henderson, 2003) (Figures S3C, S3D, and S3G) for each particle at each tilt angle. In the absence of radiation-induced rotations in the sample (and without any errors in the orientational assignments), the plot would resemble the inset in Figure S3D showing the assigned tilt angles for the particle across the 60° tilt series (the plotted dots are colored based on tilt angle from +30° [purple] to −30° [yellow]). We only selected particles from where the plots were roughly on a line (white lines in Figures S3C and S3D) to eliminate particles that were misaligned. Our analysis showed a significant departure from the ideal behavior (mean 3.8° ± 2.6° from the ideal vertical axis, Figures S3C, S3D, and S3G). In the absence of radiation-induced specimen motion, the difference in assigned in-plane shifts between particles extracted from the aligned tilt series and the corresponding sub-tomograms should be zero. However, for the selected subset of particles we observed differences in shifts with mean 5.8 ± 4.0 Å (Figures S3E and S3F). These results indicate that radiation-induced sample motions occur during acquisition of the tilt series data. These motions will violate the starting assumption of the tomographic experiment that each image in the tilt series corresponds to a well-defined projection of the same 3D structure.

### Tomographic Data Collection on Ultrastable Specimen Supports

**Figure 5:**
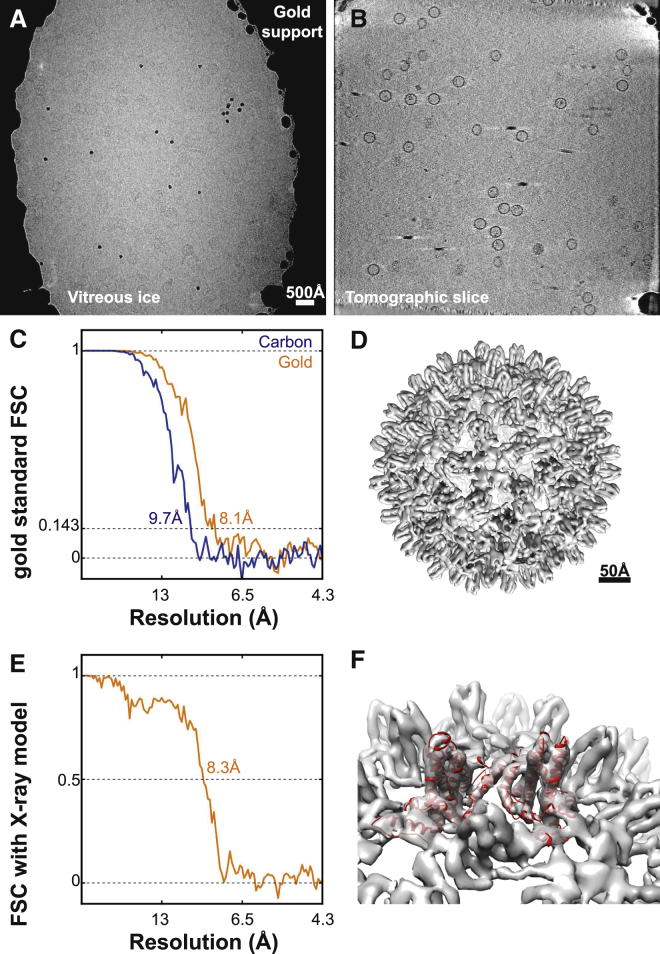
Sub-tomogram Averaging with Reduced Radiation-Induced Motion (A) Representative image from a tilt series of the hepatitis B capsid sample imaged on a gold support grid. The support is seen as a high-contrast feature. (B) Slice from a tomogram reconstructed from the tilt series data. The scale bar applies to (A) and (B). (C) FSC curves showing the estimated resolution from the refinements of data from carbon grids and gold supports. (1,145 HBV capsid particles were used in both refinements; for the gold supports these came from 11 tomograms.) (D) Isosurface representation of the output volume from the refinement with data collected on gold supports. (E) FSC curve of the sub-tomogram averaging model from the refinement with data collected on gold supports against the X-ray structure. (F) The X-ray structure is shown fitted into the sub-tomogram averaging map where secondary structure elements are clearly resolved. See also Figures S3 and S4.

Recently, specimen supports in which both the grid and the holey film are made of gold have been shown to significantly reduce radiation-induced specimen motions and improve image quality (Russo and Passmore, 2014b). To test the effects of these ultrastable grids on sub-tomogram averaging, we repeated the HBV data collection as presented in Figure 2 on gold supports (Figures 5A and 5B).

Radiation-induced sample motions were not evident upon visual inspection of the tilt series. This was confirmed by analyzing the residuals between the actual and the expected position of the fiducial markers in the aligned tilt series as calculated in IMOD. Comparison of these values with those from the data collected on traditional carbon support grids indicated that movement of the fiducial particles was reduced by nearly a factor of two on the gold supports (Figure S4). The overall average residual on the gold supports was 2.3 Å (over 11 tilt series), compared with 4.3 Å (over 15 tilt series) on traditional carbon support grids. The observed average ice thickness in tomograms from the carbon and gold support datasets was 164 ± 44 and 165 ± 25 nm, respectively. For tilt series collected on carbon support grids, defoci ranged from −3.2 to −5.6 μm with a mean of −4.4 ± 0.6 μm. The corresponding range for gold supports was −3.3 to −5.6 μm with a mean value of −4.1 ± 0.7 μm. Based on these measurements, the ice thickness and defocus values are equal, within error, for the two experiments, which entails that they cannot explain the improvement seen on the ultrastable gold substrates. Using data collected on gold supports, application of the sub-tomogram averaging procedure with the weighted 3D CTF model described above yielded a higher-resolution map (8.1 Å) from fewer particles than before (1,145 instead of 1,851, Figures 5C–5F). Together, these results show that radiation-induced motion of the specimen during tilt series acquisition is at least one of the limitations on the resolution of cryo-ET.

## Discussion

In this study, we have developed and applied an approach to sub-tomogram averaging whereby we used recent advances in single-particle cryo-EM. Our approach complements previous reports (Bartesaghi et al., 2012; Förster et al., 2005; Schur et al., 2015) and describes an alternative to resolve high-resolution details from cryo-ET data. We extended the regularized likelihood refinement algorithm in RELION to 3D images, and used ultrastable gold supports to reduce radiation-induced sample movements. Using sub-tomogram averaging on an HBV capsid sample, we demonstrated that this approach allows sub-nanometer resolution structure determination of biological macromolecules.

The extension of the (empirical) Bayesian approach in RELION to 3D data also led to a new 3D CTF model. This model expresses both phase and amplitude data for the information transfer at every point in the 3D Fourier transform of each individual sub-tomogram. Thereby, it may be used to model the missing wedge (or the geometry of the tilt series in general); variations in defocus over the tilt series; and the loss of signal in higher-tilt images. We do note that the icosahedral symmetry of the HBV capsids facilitates missing-wedge correction, and tests on particles with lower symmetry will be needed to demonstrate the full potentials of the 3D CTF model. In addition, we used knowledge about radiation-induced damage from single-particle analysis experiments to down-weight the contribution of images with higher accumulated dose in our 3D CTF model. To our knowledge, this is the most comprehensive CTF model used in sub-tomogram averaging to date. To make full use of the new capability that this model provides for CTF correction, we used a previously described extended tilt series acquisition scheme (Eibauer et al., 2012) that allows accurate CTF estimation for all images in the tilt series, even at high tilt angles. Without CTF correction, averaging of HBV sub-tomograms resulted in a featureless structure, whereas the new model outperformed the conventional strip-based phase flipping approach (Fernandez et al., 2006).

Nonetheless, our experiments with a combined approach on the same sample illustrated that single-particle analysis yields significantly higher resolutions than sub-tomogram averaging. Recording the pre-illuminated image prior to the tilt series leads to an additional dose of 6 e^−^/Å^2^ in the latter that would not be present in a conventional tomography experiment. However, Allegretti et al. (2014) observed that for F_420_-reducing [NiFe] hydrogenase, near-atomic resolution reconstructions with convincing side-chain densities could still be calculated from single-particle images that were pre-irradiated with a much higher dose than 6 e^−^/Å^2^, and similar observations were made for β-galactosidase by Bartesaghi et al. (2014). Therefore, increasing the total dose in the sample after tilt series acquisition from 60 to 66 e^−^/Å^2^ by pre-illuminating the sample in our experiment (while also down-weighting later images using the *B*–factor weighting as in Figure 1F) is not likely to explain the difference between the 10.5 Å resolution for the sub-tomogram average and the 6.5 Å resolution for the single-particle reconstruction. Comparison of the orientations assigned to the sub-tomograms and the single-particle projections, and a reconstruction from the sub-tomograms with the orientations as determined from single-particle analysis, showed that it is also not likely that errors in the sub-tomogram alignment limit the resolution of the sub-tomogram averaging approach. Therefore, we hypothesize that the quality of the tomograms themselves is a limiting factor for the resolution of the sub-tomogram average, although we cannot rule out that residual errors in our CTF correction and/or radiation damage weighting scheme also play a role.

In single-particle analysis, radiation-induced sample motion has been observed to limit resolution of the reconstruction, but movie-processing algorithms may be employed to partially correct for these movements (Bai et al., 2013; Brilot et al., 2012; Li et al., 2013; Scheres, 2014). Although using similar movie-processing algorithms may correct motions within each image of a tomographic tilt series, correction for sample movement from one tilted image to the next is currently not possible. In many tomography experiments, the gold fiducial markers used to align the tilt series have been observed to undergo radiation-induced movement during data acquisition. If only the fiducial markers moved but not the objects of interest, this movement would deteriorate the tilt series alignment, but the underlying assumption of a constant 3D macromolecular or cellular structure in the field of view would still be fulfilled. In such a case, given enough fiducial markers one would still be able to reconstruct high-resolution information in the tomograms. However, our experiments in which we tracked the radiation-induced motion of individual DNA-origami particles throughout the tilt series show that the macromolecular complexes of interest also move during the tilt series acquisition. We applied an overall dose of 70 e^−^/Å^2^, and even after alignment of the tilt series using fiducials we observed average radiation-induced translations of ∼6 Å and radiation-induced rotations of several degrees. This movement will break the basic assumption of the tomographic reconstruction, and will limit the high-resolution information content of the tomogram, even if one were to align the tilt series using the molecules of interest instead of fiducial markers (Iwasaki et al., 2005; Zhang and Ren, 2012).

Radiation-induced specimen motion has long been known to limit high-resolution information transfer in cryo-EM images (Brilot et al., 2012; Downing, 1988; Henderson and Glaeser, 1985; Wright et al., 2006). Since the bulk of this motion occurs perpendicular to the plane of the grid, its detrimental effect is expected to be much larger for the tilted images in a tomography experiment than for the untilted projections in single-particle analysis. Because correction of radiation-induced motion from one tilted image to the next would be difficult, we sought to reduce this motion experimentally by the use of ultrastable gold supports (Russo and Passmore, 2014b). Compared with traditional copper grids with a carbon film, large specimen motions perpendicular to the support are reduced by a factor of 50, while smaller motions parallel to the support are reduced more than 2-fold. Improved residuals for the fiducial marker alignment show that radiation-induced motion is indeed reduced on the gold supports. The observation that we obtained a higher-resolution map from fewer sub-tomograms compared with the carbon support grids suggests that the reduced motion resulted in tomogram reconstructions with better high-resolution information. Although the hepatitis B sample in our experiment is a single-particle-like sample, we expect that the reduced radiation-induced motions on gold supports will also improve cellular tomography.

To summarize, in this work we aimed to harness recent advances that have revolutionized single-particle cryo-EM, namely regularized likelihood refinement, direct electron detection, and ultrastable gold supports, to improve 3D structure determination by sub-tomogram averaging. Using simplified test samples, we show that full CTF phase and amplitude correction improves sub-tomogram averaging, that radiation-induced motions during tilt series acquisition may be a limiting factor, and that data collection on gold supports significantly improves resolution in sub-tomogram averaging. Although the resolution we obtain from sub-tomogram averaging still does not rival the resolution that is possible using the single-particle approach, this study is a step forward and contributes to our understanding of the remaining issues in tomography.

## Experimental Procedures

### Sample Preparation

HBV protein and DNA-origami molecules were purified as described previously (Bai et al., 2012; Wynne et al., 1999). In each case, the purified sample was mixed with 10-nm gold beads conjugated to protein-A (CMC, Utrecht) and applied to freshly plasma-treated holey grids (200 mesh Cu/Rh, carbon support Quantifoil grids with 2 μm hole size, or 300 mesh gold supports with 1.2 μm holes [Russo and Passmore, 2014b]). A higher concentration of fiducials was used in the case of the gold supports. Grids with 3 µL of the applied mixture were blotted for 2 or 5 seconds using a Whatman 595 filter paper, plunge-frozen in liquid ethane using a Vitrobot (FEI), and stored in liquid nitrogen until further investigation.

### Electron Cryotomography

Vitrified samples were imaged using a FEI Titan Krios microscope operated in EFTEM mode at 300 kV with a 70-μm C2 aperture and a 70-μm objective aperture. Cryo-ET data were collected using SerialEM (Mastronarde, 2005) on a K2 summit direct electron detector fitted behind an energy filter (Gatan Quantum) at a nominal magnification of 53,000× with a calibrated pixel size of 2.17 Å. The energy filter was set to remove electrons > ±10 eV from the zero loss peak energy. The K2 summit camera was operated in counting mode at a dose rate of ∼5–8 electrons/pixel/s on the camera except for the pre-illuminated images (in Figure 4A) where super-resolution mode was used. Each tilt image was dose-fractionated into three image frames, each with ∼0.5 e^−^/Å^2^ electron dose, and aligned during the acquisition itself using the “Combined Filter” in the Gatan Digital Micrograph software. Based on Thon ring appearance, we observed that this alignment becomes unreliable for high tilt angles. Therefore, we did not use frame alignment for tilt angles higher than 45°. For the HBV capsid sample, tilt series data were collected between ±60° with 3° tilt increments. Data were collected at 3.2–5.6 μm underfocus with a cumulative dose of 60 e^−^/Å^2^ equally fractionated over the tilt series. For DNA-origami molecules, data were collected between ±30° with 5° tilt increments. A defocus of −7 μm and a cumulative electron dose of 70 e^−^/Å^2^ was applied, and was equally fractionated over each tilt angle in the series.

During data collection on ultrastable gold supports, the hole was illuminated symmetrically with the center of the beam coinciding with the center of the hole, and where the beam encompassed the region of the gold support adjacent to the hole. Since the gold support scatters more electrons than the specimen, it is visible at very low electron doses. Therefore, a low-dose image (0.05 e^−^/Å^2^) was collected and used to center the hole using image shift before commencing data collection. Finally, because gold does not show Thon rings, the extra images for defocus estimation (cf. Figure 1C) were collected by imaging adjacent specimen holes on either side of the region of interest.

Fiducial particles in tilt series data were tracked automatically using IMOD (Kremer et al., 1996) and then inspected manually for errors. Corrections of tracking errors were made manually within IMOD, and the final aligned tilt series were produced in IMOD. Only in the comparison with strip-based CTF, the aligned tilt series data were CTF corrected by phase flipping in IMOD. Tomogram reconstructions from the aligned tilt series were conducted using weighted back-projection implemented in Tomo3D (Agulleiro and Fernandez, 2011). All sub-tomogram averaging and single-particle analysis refinements were performed in RELION with icosahedral symmetry imposed for HBV capsid particles. Additional details are provided in the Approach section.

## Accession Numbers

The sub-tomogram averaging reconstruction of the HBV capsid from ultrastable gold supports (Figure 5D) has been deposited at the Electron Microscopy Data Bank with the accession code EMD-3015.

## Supplemental Information

Document S1. Figures S1–S4

Document S2. Article plus Supplemental Information
