# Supplementary material for: Advances in Single-Particle Electron Cryomicroscopy Structure Determination applied to Sub-tomogram Averaging
Source: Structure. 2015 Sep 1;23(9):1743–53. doi: 10.1016/j.str.2015.06.026 (PMC4559595; doi:10.1016/j.str.2015.06.026)
Supplement: Document S1. Figures S1–S4 [file mmc1.pdf]

Structure, Volume *23*

Supplemental Information

Advances in Single-Particle Electron Cryomicroscopy Structure Determination applied to Sub-tomogram Averaging

Tanmay A.M. Bharat, Christopher J. Russo, Jan Löwe, Lori A. Passmore, and Sjors H.W. Scheres

Supplementary Data

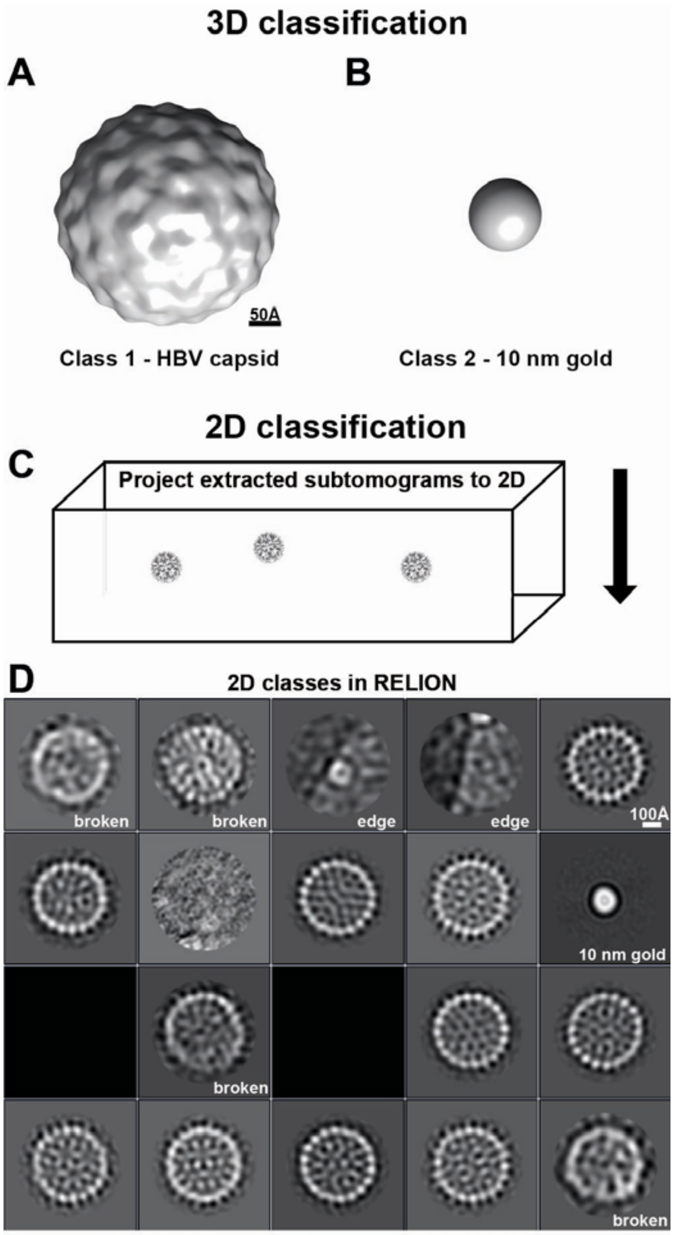

Figure S1. Initial classification of the data. (related to figure 2)
(A-B) Reference-free 3D classification of the data set was conducted. The classification clearly distinguished between extracted 10 nm gold fiducials and *bona fide* HBV capsid particles. (C) As an independent approach to classification, rather than extracting a 3D sub-tomogram, we extracted a 2D projection of each sub-tomogram. (D) These projections were used for 2D-classification in RELION. Classes showing broken HBV capsids, and classes that did not correspond to HBV capsids are marked.

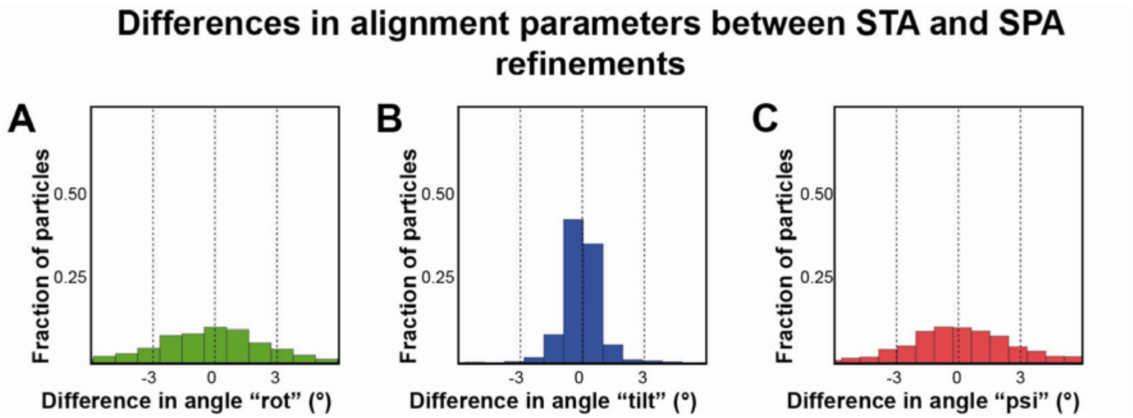

Figure S2. Histograms of differences in alignment parameters between the sub-tomogram averaging and single-particle analysis refinements. (related to figure 4) (A) A histogram of differences in the assigned first Euler angle (“rot” in RELION) between the sub-tomogram averaging and single-particle analysis refinements presented in Figure 4. (B) Corresponding histogram for the second Euler angle “tilt”. (C) Corresponding histogram for the third Euler angle “psi”.

Radiation-induced motion during tilt-series data collection

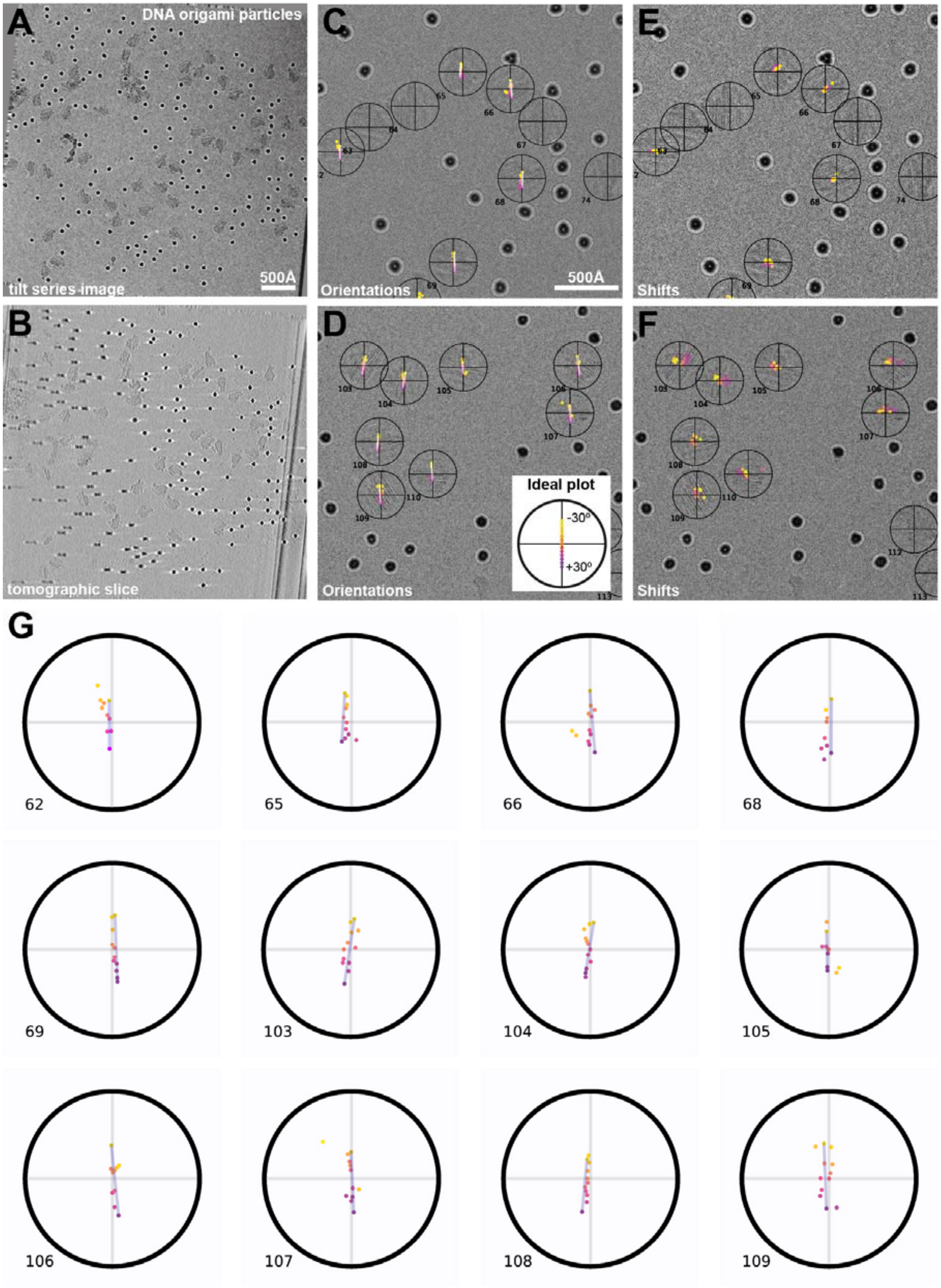

Figure S3. Radiation-induced motion occurs during tilt series data collection. (related to figure 5)

(A) A representative image from a tilt series of DNA origami molecules. Particles are large and asymmetric, allowing accurate assignment of orientation angles. The scale bar applies to panels A-B. (B) Slice from a tomogram reconstructed from the tilt series data. (C-D) Using the Euler angles from sub-tomogram averaging as a reference, a tilt-pair like analysis (Rosenthal and Henderson, 2003) was conducted for each particle at each tilt angle, and the result is shown as a polar plot made directly on the 0º image of the tilt series. The grid was tilted at 5° increments between ±30º during tilt series data collection. Each plotted dot is coloured from purple to yellow representing the order in which the data was collected from +30º (purple) to -30º (yellow) tilt of the specimen stage. In the absence of radiation-induced motion, the plot would resemble the schematic shown in the inset of D. However, significant deviations from ideality were observed. Two representative examples of tilt series images are shown. Only particles where the plotted points were roughly on a line (white lines) were used to measure differences in Euler angles to prevent quantification of mis-aligned particles. The scale bar applies to panels C-F. (E-F) Differences in Δx, Δy shifts between the selected sub-tomogram averaging alignment parameters and the single-particle analysis alignment parameters plotted onto the zero degree image of the tilt series for each particle at each tilt angle, scaled by multiplying each shift by five (the two images shown are from different tilt series). The colour of the dots in E-F is the same as in C-D. (G) Enlarged versions of the plots in panels C-D. Plotted dots have the same colour scheme as C-D. A blue line segment connecting the first and last dot is shown (white lines in panels C-D).

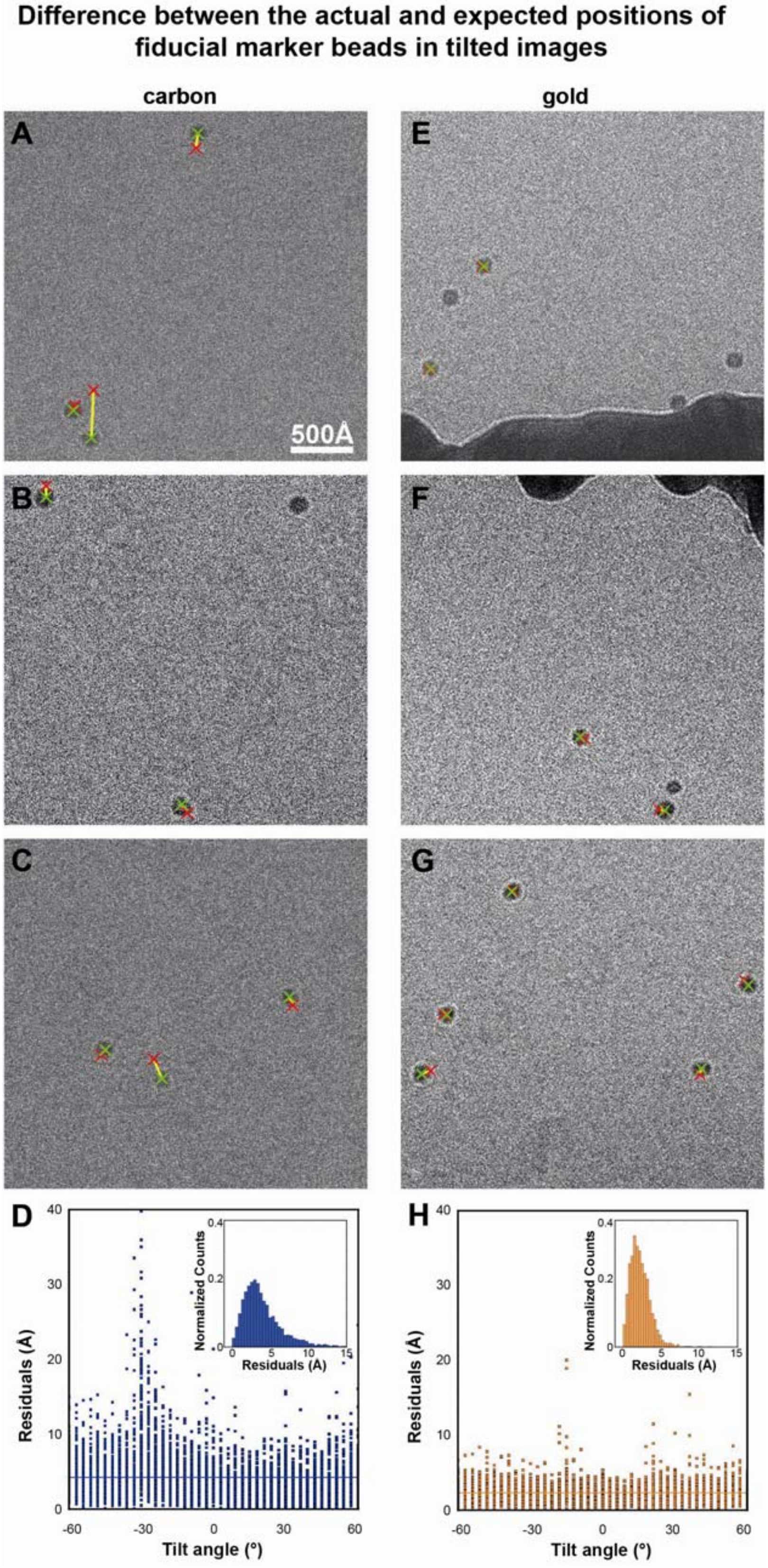

Difference between the actual and expected positions of fiducial marker beads in tilted images
carbon
gold
A
B
C
D
E
F
G
H
500Å
Residuals (Å)
Tilt angle (°)
Normalized Counts
Residuals (Å)

Figure S4. Radiation-induced movement of fiducial markers is reduced on gold supports. (related to figure 5)

(A-C) Three examples of the actual positions of 10 nm gold fiducial markers (green cross) and their expected positions (red cross) in 60° tilted images of tilt series collected on traditional carbon support grids have been shown in an IMOD residual plot. Plotted positions for the same gold fiducial marker are connected with a yellow line. The scale bar in A applies to panels A-C, E-G. (D) The overall average residual over 15 tilt series (total 5452 residuals) collected on carbon support grids was 4.3 Å (horizontal blue line). Tilt series data collection was started at -30° tilt and data was collected in two directions. Inset: histogram of residuals on carbon support grids. (E-G) Three examples of the corresponding plot for data collected on gold supports. (H) The overall averaged residual over 11 tilt series (total 3344 residuals) collected on gold support grids was 2.3 Å (orange line). Tilt series data collection was started at 0° tilt and data was collected in two directions. Inset: histogram of residuals on gold support grids.
